# Patient blood management: A solution for South Africa

**DOI:** 10.7196/SAMJ.2019.v109i7.13859

**Published:** 2019-06-28

**Authors:** J Thomson, A Hofmann, C A Barrett, A Beeton, G R M Bellairs, L Boretti, M J Coetzee, S Farmer, M W Gibbs, H H Gombotz, C Hilton, C Kassianides, V J Louw, C Lundgren, J N Mahlangu, C B Noel, V Rambiritch, F Schneider, E Verburgh, P-L Wessels, P Wessels, R Wise, A Shander

**Affiliations:** 1 South African National Blood Service, Johannesburg, South Africa; 2 Medical School and Division of Surgery, Faculty of Health and Medical Sciences, University of Western Australia, Australia; 3 Institute of Anaesthesiology, University Hospital and University of Zurich, Switzerland; 4 School of Health Sciences and Graduate Studies, Faculty of Health Sciences, Curtin University, Bentley, Western Australia, Australia; 5 International Foundation for Patient Blood Management, Basel, Switzerland; 6 Department of Internal Medicine, Faculty of Health Sciences, University of the Free State, Bloemfontein, South Africa; 7 Department of Anaesthesiology, Wits Donald Gordon Medical Centre, Johannesburg, South Africa; 8 Western Province Blood Transfusion Service, Cape Town, South Africa; 9 Department of Anaesthesiology, Port Elizabeth Hospital Complex, South Africa; 10 Department of Haematology and Cell Biology, National Health Laboratory Service and Faculty of Health Sciences, University of the Free State, Bloemfontein, South Africa; 11 Department of Anaesthesiology and Perioperative Medicine, Groote Schuur Hospital and Faculty of Health Sciences, University of Cape Town, South Africa; 12 Department of Anaesthesiology, Intensive Care Medicine and Pain Management, General Hospital Linz, Austria; 13 Department of Medicine, Faculty of Health Sciences, University of Cape Town, South Africa; and Morningside Clinic, Johannesburg, South Africa; 14 Division of Clinical Haematology, Department of Medicine, Faculty of Health Sciences, University of Cape Town, South Africa; 15 Steve Biko Centre for Bioethics, Faculty of Health Sciences, University of the Witwatersrand, Johannesburg, South Africa; 16 School of Pathology, Faculty of Health Sciences, University of the Witwatersrand, and National Health Laboratory Service, Johannesburg, South Africa; 17 World Federation of Haemophilia International Haemophilia Training Centre, Johannesburg, South Africa; 18 Bleeding Disorders Unit and Clinical Haematology Service, Charlotte Maxeke Johannesburg Academic Hospital, Johannesburg, South Africa; 19 Department of General/Transplant Surgery, Groote Schuur Hospital, Cape Town, South Africa; 20 Department of Medical Oncology, Faculty of Health Sciences, University of Pretoria, South Africa; 21 Pietermaritzburg Metropolitan Department of Anaesthetics, Critical Care and Pain Management, Pietermaritzburg, South Africa; 22 Head Clinical Unit, Critical Care, Edendale Hospital, Pietermaritzburg, South Africa; 23 Discipline of Anaesthesiology and Critical Care, School of Clinical Medicine, University of KwaZulu-Natal, Durban, South Africa; 24 Department of Anesthesiology and Surgery, Icahn School of Medicine at Mount Sinai, NY, USA; 25 Team Health Research Institute, Englewood Health, Englewood, NJ, USA

## Abstract

For more than 70 years the default therapy for anaemia and blood loss was mostly transfusion. Accumulating evidence demonstrates a significant dose-dependent relationship between transfusion and adverse outcomes. This and other transfusion-related challenges led the way to a new paradigm. Patient blood management (PBM) is the application of evidence-based practices to optimise patient outcomes by managing and preserving the patient’s own blood. ‘Real-world’ studies have shown that PBM improves patient outcomes and saves money. The prevalence of anaemia in adult South Africans is 31% in females and 17% in males. Improving the management of anaemia will firstly improve public health, secondly relieve the pressure on the blood supply, and thirdly improve the productivity of the nation’s workforce. While high-income countries are increasingly implementing PBM, many middle- and low-income countries are still trying to upscale their transfusion services. The implementation of PBM will improve South Africa’s health status while saving costs.

For decades the default treatment for anaemia and bleeding was mostly blood transfusion. However, safety risks from new and re-emerging pathogens in the blood pool,^[1–3]^ significant inter- and intrahospital transfusion variability for matched patients,^[4–9]^ the high cost of transfusion therapy,^[10,11]^ and in particular the large number of risk-adjusted observational studies demonstrating that transfusion is independently associated in a dose-dependent relationship with adverse outcomes including morbidity and mortality,^[12–25]^ have led to questioning of the transfusion paradigm.^[26,27]^ Also, systematic reviews and meta-analyses of randomised controlled trials have shown either no benefit or increased risks from liberal transfusion regimens when compared with more restrictive regimens.^[28–32]^ In response to these challenges and with the objectives of better addressing the causes or underlying diseases that could potentially lead to allogeneic transfusions and offering the best choice of therapy, the new paradigm of patient blood management (PBM) has emerged.

PBM is described by the International Foundation for Patient Blood Management as ‘an evidence-based bundle of care to optimize medical and surgical patient outcomes by clinically managing and preserving a patient’s blood’.^[33]^ This is achieved by optimising erythropoiesis, minimising blood loss and harnessing or optimising the physiological reserves of anaemia while appropriate therapy is instituted, often referred to as the ‘three pillars of patient blood management’ (Fig. 1). This concept was adopted by the World Health Assembly resolution WHA63.12 in 2010.^[34]^ A similar and widely used description of PBM by the Society for the Advancement of Blood Management is ‘the timely application of evidence-based medical and surgical concepts designed to maintain hemoglobin concentration, optimize hemostasis and minimize blood loss in an effort to improve patient outcome’.^[35]^

A number of large observational PBM studies have demonstrated significantly improved patient outcomes including mortality, morbidity and average length of hospital stay,^[36–39]^ one of these being a recent ‘real-world study’ with 605 046 patients within the jurisdiction of Western Australia (Table 1).^[40]^ Also, a growing number of randomised controlled trials looking at single PBM treatment modalities showed significantly improved patient outcomes with concurrent reduction of blood component utilisation.^[41,42]^ Overall, PBM has demonstrated improved outcomes while reducing cost and/or resource utilisation (blood components) with the potential to repurpose those savings.

## PBM as a standard of care

PBM has the potential to address several healthcare-related challenges that are specific to South Africa (SA).

First, the population of SA totalled 57.8 million in 2018^[43]^ and included an estimated 17.8 million people suffering from anaemia, mostly neonates and children, women of reproductive age, and the elderly.^[44–47]^ Apart from iron deficiency anaemia, there is also a high prevalence of HIV-related anaemia, anaemia of inflammation, malarial anaemia and anaemia from intestinal parasite infestations. In hospitalised patients, the prevalence of anaemia is even higher than in the general population, and it is an independent predictor for adverse outcomes including morbidity and mortality. ^[48,49]^ A secondary analysis of the South African Surgical Outcomes Study (SASOS), a large prospective observational study of patients undergoing inpatient non-cardiac, non-obstetric surgery at 50 hospitals across SA over a 7-day period, showed that the prevalence of preoperative anaemia was 47.8% and that it was independently associated with an increase in in-hospital mortality (odds ratio (OR) 1.657, 95% confidence interval (CI) 1.055 – 2.602; *p*=0.028) and admission to critical care (OR 1.487, 95% CI 1.081 – 2.046; *p*=0.015).^[50]^ For these reasons, the implementation of PBM including anaemia management and bleeding management represents a major opportunity to sustainably improve public health in SA in respect of:

- child mortality
- maternal mortality (particularly related to peri- and postpartum haemorrhage)
- anaemia-related adverse outcomes
- blood loss and bleeding-related adverse outcomes
- transfusion-related adverse outcomes
- overall surgical and medical outcomes in hospitalised patients.

Second, the implementation of PBM would ease the pressure on the nation’s blood supply. The high prevalence of anaemia, the high prevalence of HIV with the world’s highest absolute number of cases,^[51]^ and other frequently occurring diseases have led to a particularly high rate of 20% blood donor deferrals (Dr Jaqueline Thomson, Medical Director, South African National Blood Service, personal communication, 2018). At the same time, the demand for blood components is expected to increase owing to HIV-related anaemia and the population segment aged ≥65 years that is now growing faster than previously.^[52]^ As a consequence, the prevalence of chronic disease and surgical procedures is expected to rise, with a concomitant increased demand for blood unless there are immediate and widespread practice changes.

Third, there is also an overall economic argument for the implementation of PBM. Unmanaged or poorly managed anaemia impairs the cognitive development of children and adolescents, reduces the ability to concentrate, and diminishes work productivity with losses in some countries of up to 9% of the gross domestic product.^[53]^ Programmes promoting oral iron therapy may be effective in specific subpopulations, but in patients with profound iron deficiency and iron deficiency anaemia, who require therapy for immediate correction, an appropriate dose of intravenous iron is the best option, while treatment of the underlying cause produces a cure. ^[54]^ Improvements in patient outcomes with the concomitant savings as seen in previous studies could lead to a significant decrease in bed occupation and relieve a healthcare budget under extreme pressure.^[42,55]^

## Contradictory developments: PBM for high-income countries and transfusion for the rest?

In the healthcare systems of the advanced economies, both the private and public sectors have begun to move their focus towards pre-empting the causes for transfusion through PBM rather than expanding the infrastructure to advance transfusion therapy.^[56]^ The full implementation of PBM as a bundle of care is challenging, and some institutions are therefore following the recommendation of a stepwise implementation by introducing a series of simple PBM measures.^[57,58]^ Others are choosing comprehensive change management methodologies and multi-stakeholder management to achieve relatively fast and system-wide implementation.^[40]^ The variable implementation methodologies, and also differences in hospital cultures, may explain why the implementation of PBM remains extremely variable across many of the high-income countries. ^[59]^ However, the underlying rationale for this patient-centric approach is simple and compelling: PBM is not only associated with a better outcome, but also with less cost.^[36,38,40]^ Consequently, PBM keeps growing while blood services are experiencing a significant decline in blood utilisation and therefore re-engineer, downsize operations, or merge with other regional blood services.^[56,60]^

In contrast, in many emerging and frontier economies, the focus of health authorities is still on modernising blood services to match quality levels comparable to those in the advanced economies and on establishing additional blood centres.^[61–70]^ This expansion results in significant new investments and adds to the already large expenses of maintaining these services. In view of the poor cost-effectiveness of blood safety interventions and of transfusion therapy,^[71–73]^ it is inappropriate use of limited financial resources. The decision to allocate more resources to supporting the product-focused transfusion model rather than the patient-centric PBM model could be likened to the establishment of landlines in a geographical area bare of communication infrastructure rather than setting up highly effective and much less costly mobile phone networks *de novo*.

In view of the burgeoning clinical data on adverse transfusion outcomes, the ongoing blood safety issues, the pervasive blood sourcing problems and the overall health-economic constraints, it would be both unethical and paradoxical to continue to invest financial and human resources into the fading model of traditional blood banking while neglecting the implementation of PBM.^[74]^

## Seizing the opportunity to improve SA’s health status and patient safety while saving costs

With combined support from both the public and private sectors, including the professional medical societies, academia, healthcare providers, funders, blood services and patient organisations and the endorsement of SA’s National Department of Health, a guiding coalition of PBM champions should take the opportunity to establish PBM as a new standard of care that offers a win-win scenario.^[75]^

PBM has become a national priority of the Australian Commission on Safety and Quality in Health Care,^[76]^ and stakeholders in the public and private healthcare sectors of other countries have the opportunity to learn from the results of the state-wide PBM quality, safety and effectiveness initiative in Western Australia that significantly improved outcomes and achieved a sizeable cost and resource utilisation reduction.^[40]^ They may benefit from the freely available multidisciplinary clinical PBM guidelines and algorithms. ^[77–83]^ With local adaptations they may also use the European Commission’s guides for hospitals and health authorities on how to implement PBM sustainably as a national standard of care.^[84,85]^ Additional lessons can be learned from blood services in foreign countries that are now embracing implementation of PBM as one of their core activities.^[86–88]^

Compelling evidence, newly gained international experience and many freely available tools and templates are now allowing progress to be quickly achieved with a series of single, evidence-based PBM interventions, or preferably PBM pilot programmes to be started in both the public and private sectors, gradually adjusting and refining contents to local needs and finally implementing PBM as a national standard of care.

## Discussion

This article first describes why in many clinical settings the transfusion paradigm is fading while the PBM paradigm is rising. Overall, the evidence demonstrates that correcting anaemia through stimulated erythropoiesis, minimising blood loss through anaesthetic and surgical techniques, and optimising coagulation status before and during surgical or other interventions are associated with significantly better outcomes and less cost than the decades-old practice of transfusing allogeneic blood components. Then, the authors explain why implementation of PBM represents a solution to many of SA’s health, healthcare and economic challenges. PBM would effectively reduce the prevalence of anaemia, which owing to the country’s specific circumstances is higher than in many other countries. In combination with optimised bleeding and coagulation management, this reduction would have a positive effect on child and maternal mortality. Overall, based on international study results and in the context of secondary analysis of the SASOS, a large number of hospitalised medical and surgical patients would benefit from PBM. Its implementation would also decrease the demand for allogeneic blood components, thus easing the chronic pressure on the nation’s blood supply, which has a high number of donor restrictions as a result of HIV-related and other transmissible diseases. PBM would also contribute to socioeconomic improvements. Fewer complications, reduced morbidity and reduced hospital length of stay could lead to significant cost savings that could be reallocated to other areas of unmet clinical need. Optimal anaemia management, central to PBM, would also improve the cognitive development of children and adolescents, thus positively influencing productivity and the gross domestic product and the quality of life in SA.

The authors also allude to an important cultural aspect when it comes to the implementation of PBM: health authorities have traditionally focused on the supply of, not the demand for, blood. For decades, the legislation and administration of the ‘blood sector’ revolved around the concept that red blood cell transfusion is the only way of treating anaemia and blood loss. Therefore, the permanent and ubiquitous availability of safe donor blood was considered indispensable to keep clinical services operational, while the financial aspects of blood safety measures played only a minor role. However, with the introduction of evidence-based patient and disease management through PBM, other treatment options are now preferred, and in many clinical scenarios, blood transfusions are less frequently used or have even become obsolete.

Although this reversal of trends can now be observed in many modern healthcare systems, it may be challenging for health authorities to change focus, particularly when current efforts are still geared towards expansion of blood services. PBM will clearly impact a lot more on reducing the number of transfused blood components than any effort of the blood centres to increase the number of blood collections.

This article should be understood as a call to action to significantly improve the quality, safety and effectiveness of healthcare in SA. First, health authorities, medical professionals, funders and all other relevant stakeholders need to be thoroughly informed about the evidence on the multiple wins with PBM. This knowledge should lead to their acknowledgement and official endorsement of PBM and active support to implement it. Second, multidisciplinary peer-reviewed guidelines, algorithms and other useful tools for PBM, that have already been developed in other parts of the world and are freely accessible, need to be adapted to the local needs of SA. This should primarily take place with the active involvement of all the relevant professional medical societies in SA. Third, PBM pilot projects in the public and private sectors should be carried out to test and refine PBM in the current healthcare framework, and finally, PBM should be implemented as a mandatory national standard of care.

## Conclusions

The rapid implementation of PBM as a new standard of care is an urgent need. It offers the opportunity to sustainably and significantly improve the health status of the people of SA, with the potential of enormous savings for the healthcare system.

## Figures and Tables

**Fig. 1.**
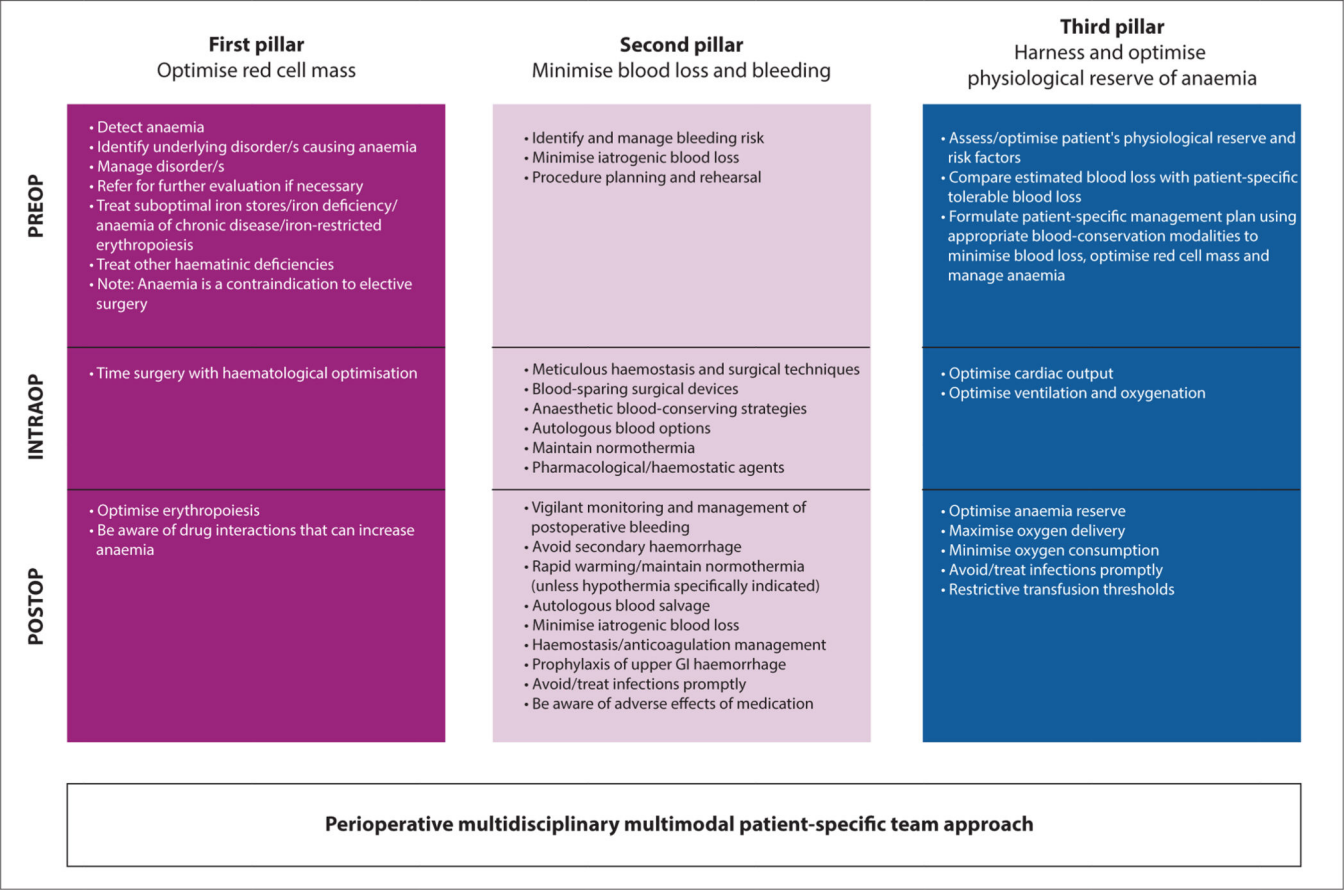
The three-pillar, nine-field matrix of PBM. This matrix was designed for the Western Australia Patient Blood Management Program to assist in the clinical implementation of the multiple PBM strategies. These strategies are considered in the perioperative period in a patient- and procedure-specific context. ^[89]^ The principles of this matrix were also applied to non-surgical patients before, during and after treatment. (PBM = patient blood management.)

**Table 1.** Summary of study results from the Western Australia Patient Blood Management Program, 2008 – 2012 (*N*=605 046), comparing baseline year (2008/09) with year 6 (2013/14) (adapted from Leahy *et al*.^[40]^)

Patient outcomes	Adjusted ratios (95% CI)	*p*-value

In-hospital mortality	0.72 (0.67 – 0.77)	<0.001
Hospital-acquired infections	0.79 (0.73 – 0.86)	<0.001
Acute myocardial infarction/stroke	0.69 (0.58 – 0.82)	<0.001
Length of hospital stay	0.85 (0.84 – 0.87)	<0.001
All-cause emergency readmission	1.06 (1.02 – 1.10)	<0.001
**Key programme indicators**	**Change**	

Preoperative anaemia	21% to 14%	0.001
Pre-transfusion haemoglobin	7.9 g/dL to 7.3 g/dL	<0.001
Single-unit transfusions	33% to 64%	<0.001
**Type of blood product**	**Reduction of units transfused**	

Red blood cells	41%	<0.001
Plasma	47%	<0.001
Platelets	27%	<0.001
**Economic indicators**		

Blood product cost reduction	AUD18.5 million	
Activity-based cost reduction (estimate)	AUD80 – 100 million	
